# Predicting and Designing Epitope Ensemble Vaccines against HTLV-1

**DOI:** 10.1515/jib-2018-0051

**Published:** 2020-01-08

**Authors:** Saruar Alam, Md. Kamrul Hasan, Omar Hamza Bin Manjur, Akib Mahmud Khan, Zinat Sharmin, Mahmud Arif Pavel, Md. Faruk Hossain

**Affiliations:** Department of Biochemistry and Molecular Biology, University of Dhaka, Dhaka, Bangladesh; Department of Biology, Florida International University, Miami, FL, USA; Department of Neuroscience, The Scripps Research Institute, Jupiter, FL, USA; St. John’s University, Department of Biological Sciences, Queens, NY 11439, USA; **Saruar Alam and Md. Kamrul Hasan:** These authors contributed equally to this article.

**Keywords:** Human T-lymphotropic virus 1, HTLV-1, Vaccine design, envelope glycoprotein GP62

## Abstract

The infection mechanism and pathogenicity of Human T-lymphotropic virus 1 (HTLV-1) are ambiguously known for hundreds of years. Our knowledge about this virus is recently emerging. The purpose of the study is to design a vaccine targeting the envelope glycoprotein, GP62, an outer membrane protein of HTLV-1 that has an increased number of epitope binding sites. Data collection, clustering and multiple sequence alignment of HTLV-1 glycoprotein B, variability analysis of envelope Glycoprotein GP62 of HTLV-1, population protection coverage, HLA-epitope binding prediction, and B-cell epitope prediction were performed to predict an effective vaccine. Among all the predicted peptides, ALQTGITLV and VPSSSTPL epitopes interact with three MHC alleles. The summative population protection coverage worldwide by these epitopes as vaccine candidates was found nearly 70%. The docking analysis revealed that ALQTGITLV and VPSSSTPL epitopes interact strongly with the epitope-binding groove of HLA-A*02:03, and HLA-B*35:01, respectively, as this HLA molecule was found common with which every predicted epitope interacts. Molecular dynamics simulations of the docked complexes show they form stable complexes. So, these potential epitopes might pave the way for vaccine development against HTLV-1.

## 1 Background

HTLV-1 is a positive single-stranded RNA virus belongs to the Retroviridae family and Delta retrovirus genus. The total genome size of HTLV-1 is 8507 nucleotides in length encoding 6 different proteins. In one stage of the HTLV-1 lifecycle, the RNA is utilized to synthesize double-stranded DNA which inserted into the genomic DNA of a host cell [1]. Retrovirus inserted into the host genome is referred to as provirus [2]. mRNA of HTLV-1 encodes different functionally crucial proteins including the gag, pol, and env genes present in other exogenous retroviruses, and several regulatory genes at the 3’ end of the genome whose products regulate the expression, splicing, and transport of the viral mRNAs. The pol gene encodes three enzyme functions: polymerase, protease, and integrase [3], [4], [5], [6], [7]. Researchers have found that in HTLV-1 infected individuals, peripheral blood mononuclear cells spontaneously proliferated in exogenous hypoxic conditions by HTLV-1 encoded Tax protein [3], [8], [9]. HTLV can be transmitted into the human in several ways, including blood transfusion, injecting drugs through parenteral routes, and through breastfeeding into children from infected mother [8], [9], [10], [11]. HTLV virus is found presently almost all over the world but most prevalent in the southern area of Japan, Africa, Oceania, and Southern America [12], [13], [14], [15], [16]. In most of the cases, HTLV-1 becomes dormant in infected individuals. HTLV-1 remains the causal agent of severe diseases that can be subdivided into three different criteria’s: Neoplasia (T cell lymphoma in adult Cutaneous, T cell leukemia), inflammatory syndromes (Uveitis, Arthropathy, HAM/TSP, Sjogren’s syndrome, T lymphocyte alveolitis, Polymyositis, Pneumopathy), and Infectious complications (Tuberculosis, Leprosy, Strongyloides stercoralis) [4], [17], [18], [19], [20], [21].

HTLV-1 preferably infects CD4^+^ and CD8^+^ T cells, therefore, the immune system elicits a T-cell mediated immunity, which binds to MHC class I or class II complexes. Activated, CD4^+^ and CD8^+^ T cells secrete cytokines asserting an immune response against HTLV-1. Cytotoxicity and the lysis of the infected cells are two concurrent events mediated by CD8^+^ T cells. However, an effective vaccine against HTLV-1 is yet to be examined. In this study, all the possible strains of HTLV-1 were considered and explored to predict an efficient vaccine against a unique envelope. Glycoprotein GP62 of HTLV-1 by using different data-driven tools, including immunoinformatics – that is one of the branches of data-driven tools that uses various sequence- and structure-based methods to solve immunological data and problems [22]. However, *in vitro* analysis is beyond our scope and must be warranted to validate our findings.

## 2 Methodology

### 2.1 Collection of HTLV-1 Specific Epitopes

Envelope Glycoprotein GP62 of HTLV-1 was targeted to design the vaccine because glycoproteins are located on the outer layer of the cell membrane and can easily be recognized by the immune system. The possibility of having an antigenic effect of this protein was validated through the Vaxijen server available at (http://www.ddg-pharmfac.net/vaxijen/VaxiJen/VaxiJen.html). Initially, immunogenic epitopes of envelope glycoprotein GP62 of HTLV-1 were collected from an online Immune Epitope Database known as IEDB (http://www.iedb.org/) [23]. A large data set has been narrowed down by the following criteria: the positive T cell assays and the human host only. Then, for MHC-I epitopes, only 9 and 10 mers have been considered as desired epitopes as different studies have ensured that most of the known epitopes processed by class I HLA are between 8 and 10 mers [24] and for MHC-II epitopes, only 15 residues containing fragments are chosen.

### 2.2 Collection and Multiple Sequence Alignment of HTLV-1-Glycoprotein B

The protein sequences in the FASTA format of epitope-bearing antigens were retrieved from the NCBI protein database (https://www.ncbi.nlm.nih.gov/protein/) and protein blast was performed through BLAST-P (https://blast.ncbi.nlm.nih.gov/Blast.cgi) against the non-redundant protein sequences (nr) database. All the protein sequences of HTLV-1 found after BLAST having E- value 0.0 were subjected to Multiple Sequence Alignment (MSA) using an NCBI tool Constraint-based Multiple Alignment (COBALT). COBALT processes consecutive multiple alignments against the query sequences of the protein. The alignment was performed through conserved pairwise constraint motifs derived from the NCBI domain database and using BLASTP, RPS-BLAST, and PHI-BLAST, respectively [25]. The result of aligned sequences was downloaded in the CLUSTAL format.

### 2.3 Variability Analysis of Envelope Glycoprotein GP62 of HTLV-1

Aligned sequences for HTLV-1-glycoprotein B were subjected to sequence variability analysis employing an online Protein Variability Server (PVS) (http://imed.med.ucm.es/PVS/) [26]. Shannon entropy (H) was selected as the variability metrics [27] and the variability threshold was set at 0.5. Afterward, only the conserved epitopes were picked and coincided entirely through their whole length.

### 2.4 Population Protection Coverage (PPC) Calculation

To trace the minimal sets of epitopes (optimal epitope combinations) with a target PPC, first, Class I binding profiles have been utilizing the IEDB class I HLA binding prediction tool available at (http://tools.immuneepitope.org/mhci/). Afterward, a class I HLA reference set has been selected at the time of prediction as these alleles were found most prevalent in the population [28]. For each MHC-II epitope, HLA II binding affinities have been predicted similarly to different alleles that prevail in the human population [29] using the IEDB class II HLA binding prediction server (http://tools.immuneepitope.org/mhcii/). To complete our dataset only epitopes having a predicted score of ANN IC50 <50 nM have been collected. Population coverage for this epitopes was calculated assigning the IEDB population coverage prediction tool available at (http://tools.iedb.org/tools/population/iedb_input) for the population of 11 regions of interest: Americas (North America, Central America, South America), Europe, Southeast Asia, West Africa, and West Indies.

### 2.5 HLA-Epitope Binding Prediction

The 3D structure of MHC class I molecule HLA-A*02:03 (PDB ID: 3OX8) and HLA-B*35:01 (PDB ID: 4PRN) were retrieved from an online Protein Data Bank server available at (www.rcsb.org/) and followed by visualization in the PyMOL software. Both HLA-A*02:03 (PDB ID: 3OX8) and HLA- B*35:01 (PDB ID: 4PRN) have been taken as a representative to analyze docking and dynamics as they are found to be involved in pressing the highest number of epitopes in the selected epitope set. Before performing a docking study, all the water molecules in the HLA protein were removed using the PyMOL. Additionally, C and F chains were removed from the HLA-A*02:03 because C and F chain come from the pre-core-protein of hepatitis b virus genotype C; C chain and acetone were removed from HLA-B*35:01. For the docking study, the ALQTGITLV and VPSSSTPL epitope were chosen because both showed the highest number of interactions with different HLAs. The 3D structure of ALQTGITLV and VPSSSTPL were predicted by the help of an online tool at the PEP-FOLD server. It predicts the* de novo* structure of a protein (http://bioserv.rpbs.univ-paris-diderot.fr/PEP-FOLD/). The Vina wizard tool of the PyRx software package (version 1.5.6) was exploited for docking study. Both the HLA protein HLA-A*02:03 and ligand (peptide epitope ALQTGITLV); HLA-B*35:01 and ligand (peptide epitope VPSSSTPL) files were firstly converted into PDB format to make them suitable for the docking analysis. The grid space of the center for binding analysis was set manually to facilitate the binding interaction between epitope and the binding groove of HLA-A*02:03 and HLA-B*35:01.

### 2.6 Molecular Dynamics Simulation

MD simulations were performed using the YASARA dynamics program [30], [31], to reveal changes at the atomic level in a different time scale of the docked ALQTGITLV-4PRN and VPSSSTPL-3OX8 complexes. Before starting the simulation, a cubic cell was formed by extending 8 Å on each side of the protein and a periodic boundary condition was maintained. The AMBER14 force field was applied for simulations [32]. The cell was filled with water at a solvent density of 0.998 gm/cm^3^. To replicate the physiological ion concentration, NaCl salt at 0.9% concentration was added to the solvent. For short-range Coulomb and van der Waals interaction, the cut-off radius was set to 8 Å. The long-range electrostatic interactions were measured by PME (particle-mesh Ewald) method [33]. MD simulations for both complexes were run for 50 ns at 310 K with a time step interval of 2.5 fs. Snapshots of the simulation were saved at every 250 ps for root mean square deviation (RMSD) and root mean square fluctuation (RMSF) analysis.

### 2.7 Prediction of B-Cell Epitope

B-cell epitopes (Linear) of the given protein sequence were predicted from the B-cell epitope prediction tool available at (http://tools.iedb.org/bcell/) as an antigenicity prediction method. Here, Kolaskar and Tongaonkar antigenicity prediction method has been selected because this method is based on a table that corresponds to the occurrence of amino acid residues is experimentally have known as segmental epitopes. This method can ensure approximately 75% accuracy in antigenic peptides prediction [34].


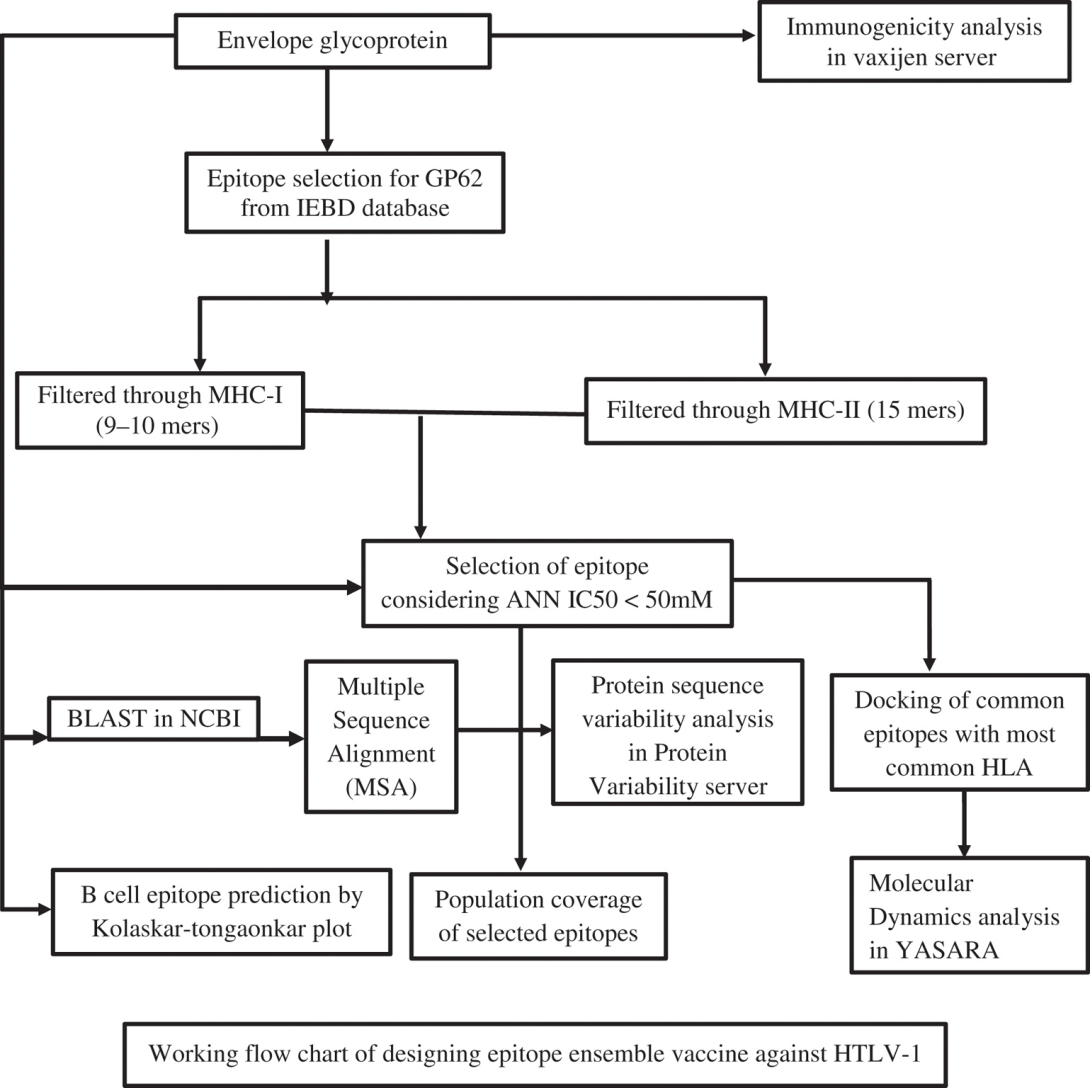


**Figure 1:**
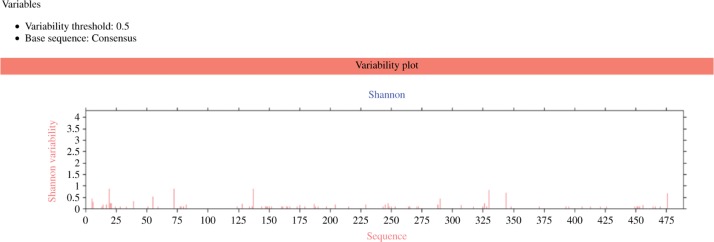
Sequence variability of Envelope Glycoprotein GP62 of HTLV-1

 The Flow Chart expounds the whole process of our study in the identification and characterization of potential epitope-based vaccines for the HTLV-1 (Figure 1).

## 3 Results and Discussions

### 3.1 Primary Selection of Epitopes from IEDB

Using the Vaxijen server we have found Envelope Glycoprotein GP62 of HTLV-1 as probable antigen under the threshold of 0.5. Vaxijen predicts antigen based on the biophysical and physicochemical properties of proteins without harboring sequence alignment [35]. For the purpose of the primary selection of epitopes of subjected protein, the IEDB database was used. We have found a total of 52 epitopes for MHC I each having 9–10 amino acids and 17 epitopes for MHC II each containing 15 amino acids (Table 1). The epitopes highlighted in green indicate the final selection against which population coverage was calculated.

**Table 1:** Selected epitopes for both MHCI and MHCII from IEDB.

Total epitopes for MHCI with 9 and 10 mers	Total epitopes for MHCII with 15 mers
DYSPSCCTL, FFQFCPLIF, HFSKCGFPF, VLYSPNVSV, ALQTGITLV, FLNTEPSQL, LPPTAPPLL, QLPPTAPPL, APGYDPIWFL, AVPVAVWLV, CFDPQIQAI, CGFPFSLLV, DLGLSQWAR, DPCSLKCPY, EPSQLPPTA, EVDKDISQL, EVSRLNINL, FLATLILFF, FPFSLLVDA, GKFLATLIL, HEVDKDISQL, ILAGPCILR, ILFFQFCPL, IVKNHKNLLK, KPNRNGGGY, LALSADQAL, LFFQFCPLI, LPHSNLDHI, LQPPCPNLV, LQSTNYTCI, LRHLPSRVR, PILQERPPL, PPCPNLVSY, PPLLPHSNL, QERPPLENRV, QEVSRLNINL, RAVPVAVWL, REALQTGITL, RPPLENRVL, SGKSLLHEV, SKLLTLVQL, SLLVDAPGY, SPCHNSLIL, TEPSQLPPTA, TLTIGVSSY, TPLLYPSLA, TQEVSRLNI, VILAGPCIL, VPSSSSTPL, WEQGGLCKA, WEQGGLCKAL, RVLTGWGLN	RGLDLLFWEQGGLCK, ALLLLVILAGPCILR, DPIWFLNTEPSQLPP, FWEQGGLCKALQEQC, GLDLLFWEQGGLCKA, KNLLKIAQYAAQNRR, LDHILEPSIPWKSKL, PSRVRYPHYSLIKPE, QHDVNFTQEVSRLNI, QLRHLPSRVRYPHYS, SQWAREALQTGITLV, SRLNINLHFSKCGFP, VILAGPCILRQLRHL, YHATYSLYLFPHWTK, HILEPSIPWKSKLLT, LEPSIPWKSKLLTLV, IAQYAAQNRRGLDLL

### 3.2 Secondary Selection of Epitopes for MHCI and MHCII Based on Predicted ANN IC50 Score

It has been exploited both the tools including, (http://tools.immuneepitope.org/mhci/) and (http://tools.immuneepitope.org/mhcii/) to analyze the binding prediction of MHC class I and MHC class II, respectively. Throughout the analysis, it has been found that only 10 epitopes having an ANN IC50 score of less than 50 among previously primary selected 52 epitopes for MHC I and 5 epitopes for MHC II out of 17 epitopes (Table 2).

**Table 2:** Selected epitopes for MHCI and MHCII based on ANN IC50 score.

Allele	Peptide	Peptide length	ANN Ic50
HLA-A*02:03	VLYSPNVSV	9	9.22
HLA-A*02:01	VLYSPNVSV	9	13.8
HLA-A*02:03	ALQTGITLV	9	4.19
HLA-A*02:01	ALQTGITLV	9	15.85
HLA-A*02:06	ALQTGITLV	9	35.51
HLA-A*02:03	FLNTEPSQL	9	5.34
HLA-A*02:06	AVPVAVWLV	9	16.26
HLA-B*35:01	DPCSLKCPY	9	43.06
HLA-B*35:01	FPFSLLVDA	9	49.62
HLA-B*35:01	LALSADQAL	9	23.95
HLA-B*40:01	REALQTGITL	10	9.88
HLA-B*07:02	RPPLENRVL	9	46.1
HLA-B*07:02	VPSSSSTPL	9	9.5
HLA-B*35:01	VPSSSSTPL	9	36.18
HLA-DRB1*01:01	ALLLLVILAGPCILR	15	4.2
HLA-DRB1*07:02	DPIWFLNTEPSQLPP	15	15.6
HLA-DRB1*04:01	DPIWFLNTEPSQLPP	15	34.6
HLA-DRB1*11:01	KNLLKIAQYAAQNR	15	37.1
HLA-DRB1*15:01	KNLLKIAQYAAQNR	15	40.1
HLA-DPA1*03:01/HLA-DPB1*04:02	LEPSIPWKSKLLTLV	15	33.5
HLA-DPA1*02:01/HLA-DPB1*01:02	LEPSIPWKSKLLTLV	15	41.3
HLA-DRB1*11:01	IAQYAAQNRRGLDL	15	40.2

### 3.3 Final Selection of Epitopes from PVS Result

Multiple sequence analysis (MSA) was followed by the analysis of the variability of Envelope Glycoprotein GP62 of HTLV-1 using a Protein variability server (PVS) where the Shannon variability method was selected for sequence variability options and a conserved fragment of length was selected, 9 with variability threshold of 0.5. Envelope Glycoprotein GP62 of HTLV-1 was consensus under a variability threshold of 0.5 (Table 3).

**Table 3:** Selected epitopes that have ANN Ic50 score below 50 and found consensus after PVS analysis.

Allele	Peptide	Peptide length	ANN Ic50
HLA-A*02:03	VLYSPNVSV	9	9.22
HLA-A*02:01	VLYSPNVSV	9	13.8
HLA-A*02:03	ALQTGITLV	9	4.19
HLA-A*02:01	ALQTGITLV	9	15.85
HLA-A*02:06	ALQTGITLV	9	35.51
HLA-A*02:03	FLNTEPSQL	9	5.34
HLA-A*02:06	AVPVAVWLV	9	16.26
HLA-B*35:01	DPCSLKCPY	9	43.06
HLA-B*35:01	FPFSLLVDA	9	49.62
HLA-B*40:01	REALQTGITL	10	9.88
HLA-B*07:02	RPPLENRVL	9	46.1
HLA-B*07:02	VPSSSSTPL	9	9.5
HLA-B*35:01	VPSSSSTPL	9	36.18
HLA-DRB1*01:01	ALLLLVILAGPCILR	15	4.2
HLA-DRB1*07:02	DPIWFLNTEPSQLPP	15	15.6
HLA-DRB1*04:01	DPIWFLNTEPSQLPP	15	34.6
HLA-DRB1*11:01	KNLLKIAQYAAQNR	15	37.1
HLA-DRB1*15:01	KNLLKIAQYAAQNR	15	40.1
HLA-DPA1*03:01/HLA-DPB1*04:02	LEPSIPWKSKLLTLV	15	33.5
HLA-DPA1*02:01/HLA-DPB1*01:02	LEPSIPWKSKLLTLV	15	41.3
HLA-DRB1*11:01	IAQYAAQNRRGLDLL	15	40.2

This plot is drawn putting the amino acid's position in the x-axis and the Shannon variability in the y-axis. A higher value in the y-axis means the higher variability in amino acid for a specific position. From those variability plots, it is easily noticeable that for Envelope Glycoprotein GP62 of HTLV-1, amino acids between 1^st^ and 25^th^ position and some of the amino acid positioned near 70, 140, 330, 345, and 475 are slightly variable. Otherwise, the overall position of amino acid positions is highly conserved. Finally, we have selected only those epitopes that specifically matched (consensus) with their full length of the generated sequences and have found a total of 9 epitopes for MHC I and 5 epitopes for MHC II that meet up these criteria.

### 3.4 Population Coverage Calculation

A combined population coverage of predicted epitopes for both the Class I and Class II MHC was calculated by using the population coverage tool (http:/tools.immuneepitope.org/tools/population) from the IEDB analysis resource. It has been a set of 14 epitopes for the calculation and interacting alleles were put against each epitope as MHC Restricted Allele(s) (Table 4). The combined calculation for both Class I and II was done in this study.

**Figure 2:**
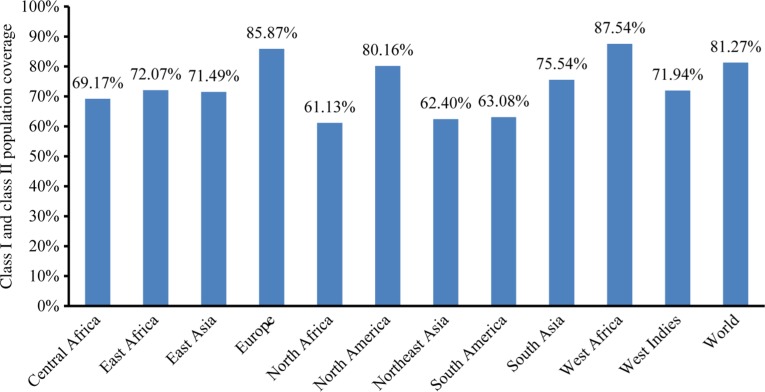
Population coverage by MHC Class I + II restricted epitopes from envelope Glycoprotein GP62 of HTLV-1

A total of 11 regions of the world and overall world coverage were calculated. Among 11 regions, 7 regions were found to have population coverage of more than 70% and overall world population coverage found to be 81.27% and the highest population coverage was found to be 87.54% and 85.87% in West Africa and Europe, respectively. From the population coverage graph, it is observable that the highest population coverage in North America is 81.28%. Central America, Europe, and West Africa also show a high population coverage which is more than 70% (Figure 2).

**Table 4:** Selected epitopes and their interacting alleles.

Number	Epitope	HLA to interact with
1	VLYSPNVSV	HLA-A*02:03, HLA-A*02:01
2	ALQTGITLV	HLA-A*02:03, HLA-A*02:01, HLA-A*02:06
3	FLNTEPSQL	HLA-A*02:03
4	AVPVAVWLV	HLA-A*02:06
5	DPCSLKCPY	HLA-B*35:01
6	FPFSLLVDA	HLA-B*35:01
7	REALQTGITL	HLA-B*40:01
8	RPPLENRVL	HLA-B*07:02
9	VPSSSSTPL	HLA-B*07:02, HLA-B*35:01
10	ALLLLVILAGPCILR	HLA-DRB1*01:01
11	DPIWFLNTEPSQLPP	HLA-DRB1*07:01, HLA-DRB1*04:01
12	KNLLKIAQYAAQNRR	HLA-DRB1*11:01, HLA-DRB1*15:01
13	LEPSIPWKSKLLTLV	HLA-DPA1*03:01/HLA-DPB1*04:02,
		HLA-DPA1*02:01/HLA-DPB1*01:02
14	IAQYAAQNRRGLDLL	HLA-DRB1*11:01

### 3.5 HLA-Epitope Binding Prediction

**Figure 3:**
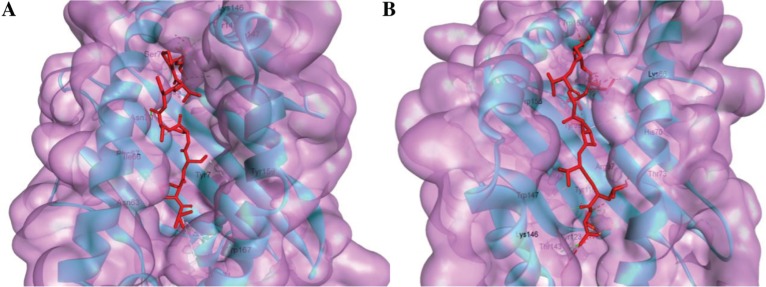
Docking predicts the interaction of predicted epitopes to MHC class I molecule, HLA-A*02:03 and HLA-B*35:01. Binding of “ALQTGITLV” to the interacting grooves of the generated structure of HLA-A*02:03 (binding energy: −8.4 Kcal/mol). Binding of “VPSSSTPL” to the binding grooves of the retrieved structure of HLA-B*35:01. (Binding energy: −8.5 Kcal/mol) The blue colored portion and green portion in both figure (A and B) represent HLA-A*02:03 molecule, ALQTGITLVL epitope and HLA-B*35:01 molecules and VPSSSTPL epitope, respectively.

PEP-FOLD was used to generate the 3D structure of the predicted epitopes. Both linear and disulfide bonded cyclic peptides containing 9–36 amino acids were allowed during the model prediction [36]. For determining the lead compounds that correspond to the desired biological function, virtual molecular screening was utilized for docking small molecules (ligands) with macromolecules [37]. Nine possible binding models are predicted as described in the methodology. Based on higher binding energy with HLA-A*02:03 and HLA-B*35:01, the best output model for ALQTGITLV and VPSSSTPL epitope was found to have a binding energy of −8.4 and −8.5 kcal/mol, respectively. The interacting and the binding of HLA-epitopes are illustrated in Figure 3.

### 3.6 Molecular Dynamics Simulation of the HLA-Epitope Docked Complexes

**Figure 4:**
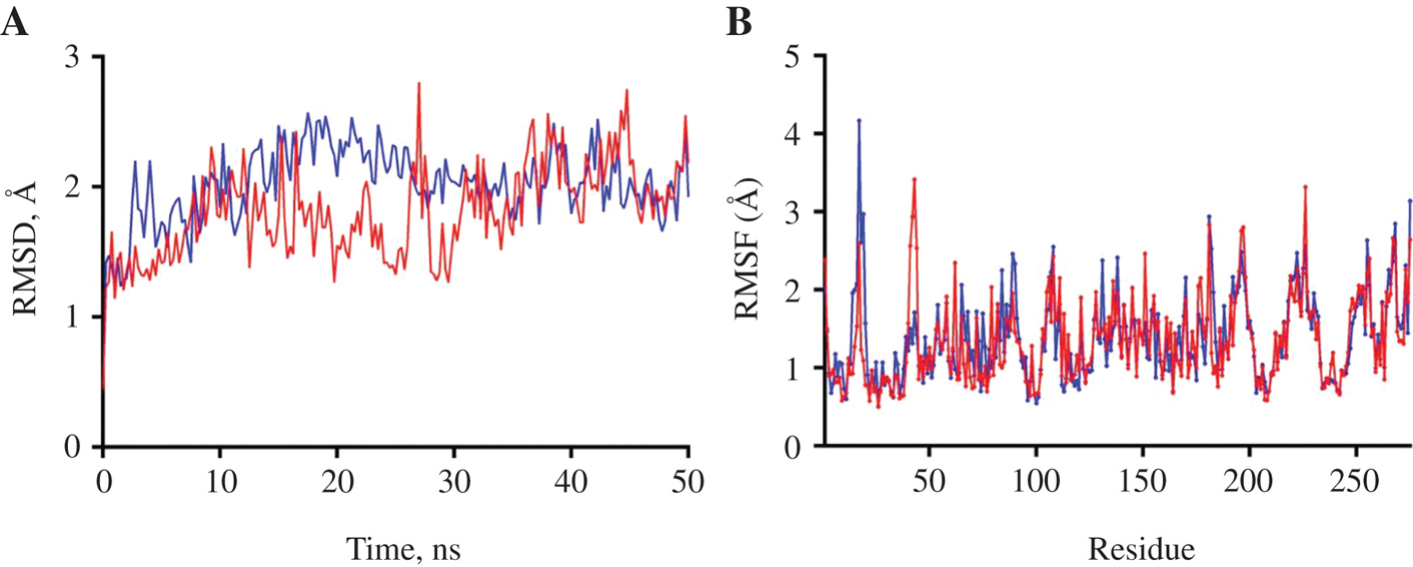
(A) RMSD values of Cα atoms and (B) RMSF values of residues in chain A of ALQTGITLV-4PRN (red) and VPSSSTPL-3OX8 (blue) complexes

**Figure 5:**
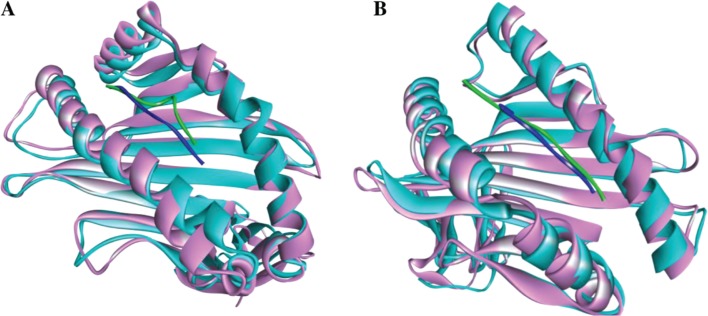
Superimposed images of (A) ALQTGITLV-4PRN and (B) VPSSSTPL-3OX8 complexes before and after 50 ns MD simulation. (Pink- protein before simulation; Cyan- protein after simulation; Blue- peptide before simulation; Green- peptide after simulation)

To understand the dynamics of the bound peptides in binding grooves of the HLA proteins within an aqueous environment, we performed 50 ns MD simulation of the docked complexes. The average RMSD values of the Cα atoms of ALQTGITLV-4PRN and VPSSSTPL-3OX8 complexes are 1.81 Å and 2.02 Å, respectively. The RMSD values for both the complexes remain relatively constant after 35 ns at around 2 Å values (Figure 4A) which suggests that the peptides and the HLA proteins are likely to form stable complexes in an aqueous environment. Moreover, the bound peptides do not show significant deviation in position after the 50 ns simulation compared to their initial docked position (Figure 5A and B). RMSF values of chain A of the HLA proteins are presented in Figure 4B. The average fluctuation value observed for most of the residues in both complexes is around 2 Å. Slightly high fluctuation values are observed in the 41–44^th^ positioned residues (2.5 Å–3.4 Å) of the ALQTGITLV-4PRN complex and also in the Arg17 (4.1 Å) residue of the VPSSSTPL-3OX8 complex. Both the 41–44^th^ residues and the Arg17 residue are located in the beta-beta joining loops of the MHC peptide-binding site. One possible reason for the fluctuation observed in these regions could be due to the adjustment of the beta-sheets in the binding site to better accommodate the peptides.

### 3.7 B Cell Epitope Prediction

**Figure 6:**
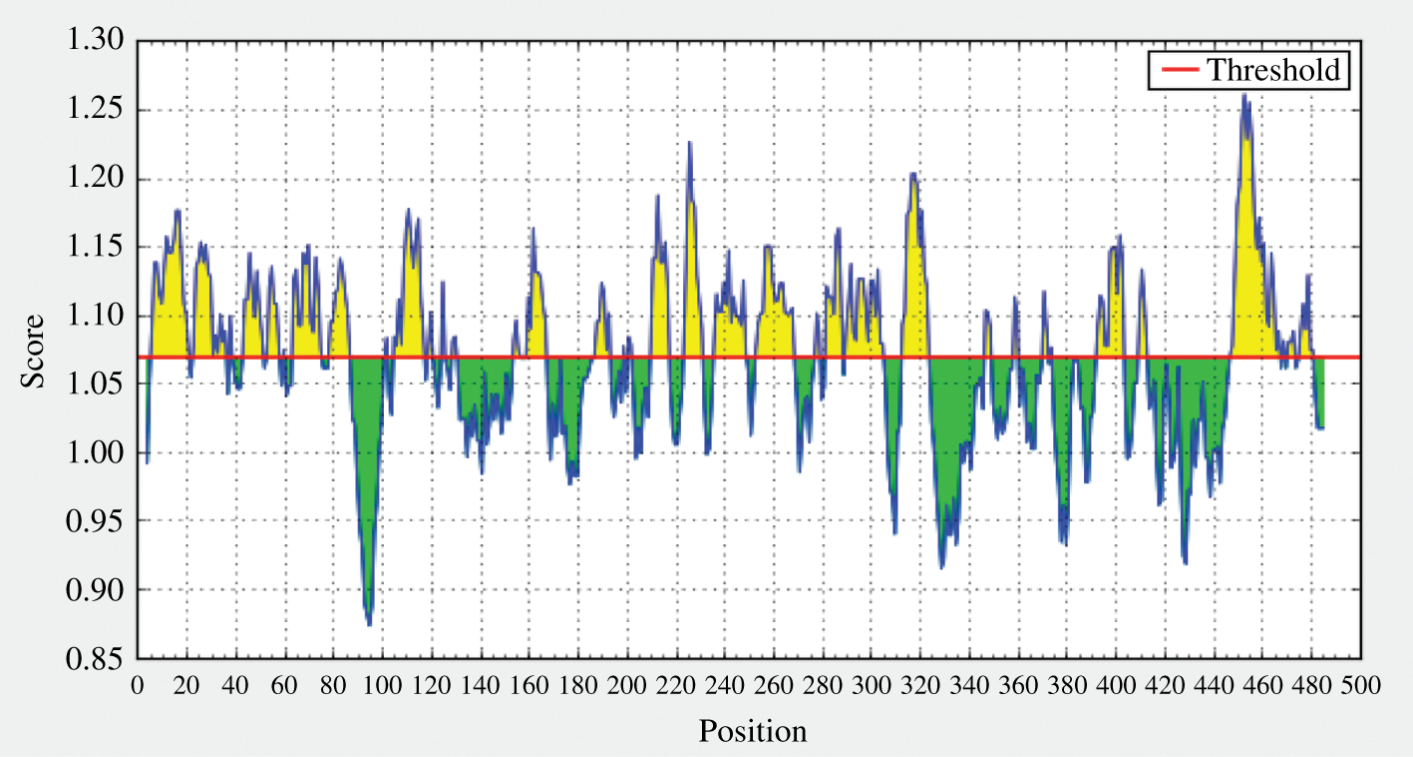
Predicting linear B-cell epitope by using Kolaskar and Tongaonkar prediction tool. Here, the picks rise above the threshold sequence can act as B-cell epitope to induce humoral immune response. The average score of the antigen gp62 of HTLV-1 is found to be 1.069.

To show whether the Envelope Glycoprotein GP62 of HTLV-1 elicits B cell immunity we have employed Kolaskar and Tongaonkar prediction method based on the physicochemical properties of amino acids in the proteins and the abundances in the experimentally known epitopes [34]. The amino acid position is represented by the x-axis, and the antigenic propensity of the protein is represented by the y-axis. The average antigenic propensity was found to be 1.069. So, all the residues having a value of greater than 1.069 are potential antigenic determinant (Figure 6).

Nineteen peptides (Table 5) are found to be a potential antigen that satisfies the set threshold value. The peptide “ITLVALLLLVILAGPCILRQL” ranging from 447 to 467 amino acid residue is predicted to have the highest antigenic propensity score.

**Table 5:** Predicted Linear B cell peptides in the envelope glycoprotein gp62 of HTLV-1.

No.	Start	End	Peptide	Length
1	6	21	ATLILFFQFCPLILSD	16
2	23	36	SPSCCTLTIGVSSY	14
3	44	51	AQPVCSWT	8
4	53	58	DLLALS	6
5	64	75	QPPCPNLVSYSS	12
6	79	87	TYSLYLFPH	9
7	105	117	SDPCSLKCPYLGC	13
8	160	167	PFSLLVDA	8
9	188	193	PPLLPH	6
10	210	217	LLTLVQLT	8
11	224	231	TCIVCIDR	8
12	236	249	TWHVLYSPNVSVPS	14
13	253	268	TPLLYPSLALPAPHLT	16
14	282	288	QAIVSSP	7
15	290	305	HNSLILPPFSLSPVPT	16
16	313	323	AVPVAVWLVSA	11
17	393	403	CKALQEQCCFL	11
18	447	467	ITLVALLLLVILAGPCILRQL	21
19	476	481	YPHYSL	6

## 4 Conclusion

Our study has predicted 14 epitopes that show good MHC binding profile, as well as, the overall population coverage was found good enough to make them suitable candidates. Using the predicted peptides and addition of potential molecule(s), if required, to enhance the immunogenicity might pave the way to designing a universal epitope-based vaccine against HTLV-1. Furthermore, collaboration with a wet laboratory is important to identify and validate the best peptide among the candidates as a peptide-based vaccine.
